# Case report: a diagnostically challenging conjunctival mass caused by the Epstein-Barr virus

**DOI:** 10.1186/s12886-015-0111-2

**Published:** 2015-10-07

**Authors:** Jordan V. Chervenkoff, Saul N. Rajak, Paul G. Brittain, David A. Wright, Victoria J M Barrett

**Affiliations:** Sussex Eye Hospital, Eastern Road, Brighton, BN2 5BE UK; Royal Sussex County Hospital, Brighton, BN2 5BE UK

## Abstract

We present a paediatric case of infectious mononucleosis in a 13-year old, manifesting with follicular conjunctivitis and a conjunctival mass in one eye with no evidence of leucocytosis on the blood count. The diagnosis was confirmed following surgical excision and biopsy. The case represented a diagnostic challenge due to its atypism and given the steady increase in the prevalence of EBV-related ocular diseases in the last years, this report can serve as an example to prompt earlier serological tests to identify the aetiology in similar cases. This is important because EBV can be treated with acyclovir early in the active viral phase.

## Introduction

The Epstein-Barr virus (EBV) is a double-stranded DNA human herpes virus type 4 (HHV-4) [1]. It is transmitted mainly through oral secretions and establishes latency by invading memory B-cells, resulting in a high prevalence throughout the world (about 90 % of adults are lifelong carriers) [2, 3]. Primary EBV infection generally occurs asymptomatically in the early years of life but in adolescence its hallmark is infectious mononucleosis (IM) [4, 5]. Viral proliferation induces both cellular and humoral immunologic responses and there is an increase in the overall number of mononuclear lymphoid cells. Thus, the condition usually presents with malaise, sweats, lymphadenopathy, fever and pharyngitis [6]. Blood results often demonstrate marked leucocytosis with a high differential lymphocyte cell count and atypical enlarged lymphoid cells.

In ophthalmology, EBV has been implicated as a causative agent in various ocular malignancies and infections which affect mostly the anterior segment (as a whole EBV-related ocular diseases have been on the rise in the past decade) [7–9]. The virus has a preference for mucosa-associated lymphoid tissues (MALT), which are rich in B lymphocytes, such as the conjunctiva or the lacrimal glands [9–11].

In this article we present a case of an EBV-related follicular conjunctivitis with an unusual unilateral bulbar and subtarsal mass in a paediatric patient, which was diagnostically challenging due to the atypical blood picture.

## Case report

A 13-year old boy presented to the accident and emergency with a large painless conjunctival mass under the right upper eyelid. It had been first noticed two weeks previously in the right supranasal quadrant and had gradually increased in size since then. No itching or excessive lacrimation were reported. The child had had a febrile illness and had been complaining of malaise, intermittent chills and sore-throat in the past 4 days. Otherwise, no significant past medical or ophthalmic history was reported and he was not on any medication. In addition, there were no known allergies.

**Fig. 1:**
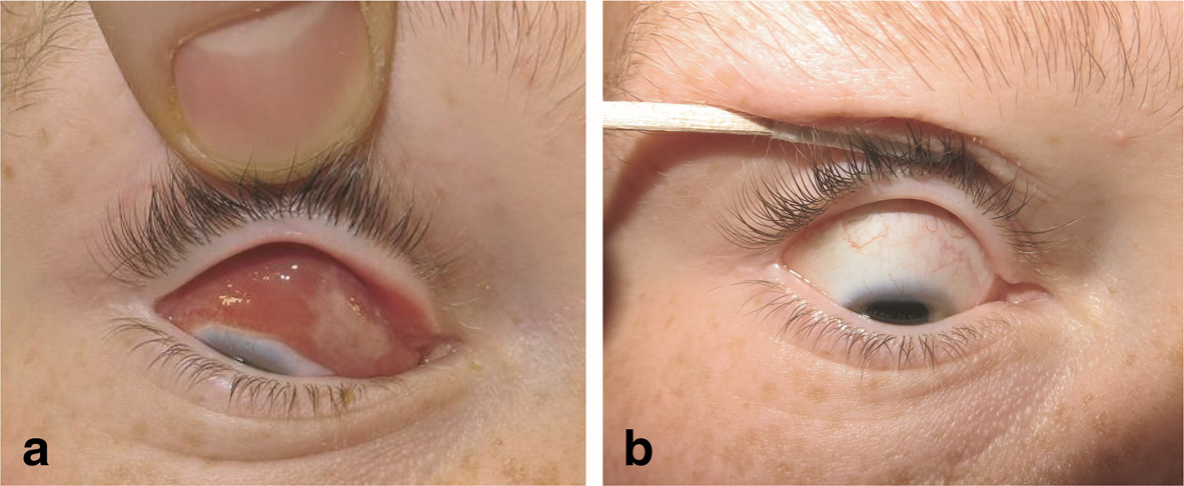
Pre-operative extend of lesion (**a**) and post-operative outcomes (**b**)

On examination, there was no pain during extraocular movements (EOM), no proptosis or diplopia. Vision was normal (Snellen acuity of 6/9) in both eyes. The intraocular pressure (IOP) was 18 mmHg bilaterally. Conjunctival follicles were present on the upper and lower eyelids in both eyes. The mass was obvious on lid retraction involving both the bulbar and subtarsal conjunctiva. It was salmon-pink, non-tender and spread across the entire conjunctiva superior to the limbus with no corneal involvement, as seen on Fig. 1a. It had well delineated borders. Pupils were equal and reactive to light. The anterior chamber was deep and clear and no abnormalities were detected in the posterior segment.

The boy’s temperature was 37.9 °C. One lymph node was palpable on the left and two on the right–all in the upper cervical chain. They were mobile and non-tender. There was no organomegaly on palpation.

Blood tests indicated that serum alanine transaminase (ALT) was elevated at 60 IU/L (normal range: 0–41 IU/L) and haemoglobin was slightly low at 131 g/L (normal range: 135–180 g/L). The overall and differential white blood cell counts (WBC) were normal, as illustrated on Table 1.

**Table 1:** Total leucocyte and differential cell counts

WBC: 5.9 × 10^9^/L (normal range: 4.0–11.0)
Differential:
Neutrophils 2.0 × 10^9^/L (normal range: 2.0–7.5) 34.1 %
Lymphocytes 3.2 × 10^9^ (normal range: 1.3–3.5) 54.5 %
Monocytes 0.6 × 10^9^ (normal range: 0.2–0.8) 9.9 %
Eosinophils 0.0 × 10^9^ (normal range: 0.0–0.4) 0.5 %
Basophils 0.1 × 10^9^ (normal range: 0.0–0.1) 1 %

A successful excision was performed under general anaesthetic and the mass was sent for a biopsy. Post-operative results at 7 days were excellent (Fig. 1a and b). The general symptoms had subsided and the child had regained his normal function. There was a small residual lymph node palpable on the left in the upper cervical chain.

## Discussion

Diagnosing the lesion was challenging and initially two types of malignancies were considered: rhabdomyosarcoma and lymphoma. Conjunctival MALT lymphoma is the commonest malignant orbital tumour and it is characteristically described as a “salmon-patch” on the conjunctiva often arising from the fornix [12]. However, it characteristically presents later in life than the age of the patient (median 65 years) [13]. On the other hand, rhabdomyosarcoma is a common primary orbital malignancy that can present with conjunctival swelling starting in the superonasal segment [14]. The majority of cases are observed in the first 10 years of life and progress rapidly giving rise to visual disturbances due to proptosis or limited EOM [14, 15]. Other differentials to consider include malignant processes such as ocular infiltration secondary to multiple myeloma or infective causes such as nodular anterior scleritis, chlamydia, herpetic infection, papilloma, tuberculosis and migratory phlyctenulosis [16].

The histological report of the excised mass demonstrated a population of large atypical centroblastic CD20 positive cells. These large proliferating B cells are representative of EBV in the active latent stage [17]. A T-cell mediated response against the rapidly dividing B cells usually occurs in non-immunocompromised individuals [18]. Hence, the conjunctival enlargement was the result of the accumulation of histiocytes, pleomorphic lymphoid and plasma cells. Additionally, thirty to forty percent of both B and T cell nuclei expressed Epstein-Barr encoded RNAs (EBERs). Multiple polyclonal cell populations were detected by polymerase chain reaction (PCR) looking for TCR or IGH gene rearrangements. This was in keeping with a reactive, rather than a neoplastic process [19].

Serology revealed the presence of Epstein-Barr nuclear antigen (EBNA) immunoglobulin G (IgG) and EBV capsid antigen immunoglobulin M (IgM) antibodies. Viral EB DNA was detected at low levels at less than 1000 copies/ml. This serological profile was consistent with the histological findings of a recent acute immunoreactive process against EBV. Notably, the presence of EBNA IgG was indicative of the virus establishing latency following primary infection (the serological genome detection assay was performed 42 days after the initial presentation) [5].

The case we presented was challenging because lymphocytosis (a characteristic feature of IM) was not evident on the full blood count at initial presentation which was about two weeks after the onset of symptoms. Normally a relative or absolute increase in the number of lymphocytes (with a 15–25 % increase in atypical cells) would be expected within the first 14 days [20]. Due to the cross-reactivity of some of the antibodies, the Monospot test can be used to confirm the diagnosis [20]. Its specificity is close to 100 % (96–100 %), however because about 10 % of people do not produce heterophil antibodies it is less sensitive (70–92 %) [21]. A clinical picture highly suggestive of IM in addition to a positive Monospot test can be used to exclude other causes of infectious mononucleosis such as: cytomegalovirus (CMV), herpes symplex, Toxoplasma gondii and human immunodeficiency virus type I (HIV-1).

Retrospectively, the elevated ALT presented a clue due to the characteristic elevation of liver transaminases in IM [22]. As a difference, alkaline phosphatase and gamma-glutamyl transpeptidase levels do not usually change in IM.

Young patients with suspicious lymphoid periocular lesions with a recent history of a febrile illness, malaise, lymphadenopathy, pharyngitis and other presenting features of IM should be offered a full blood count and serological studies to check for recent EBV infection or reactivation. If the aetiology is viral and depending on the extent of the mass, surgical management can be offered, as in our case. Otherwise, acyclovir can be used because the EB virus is susceptible to it in the active phase. Moreover, there have been reports where spontaneous regression of a smaller nodule of similar origin was observed, which demonstrated that conservative treatment may also be an option [23].

## Summary points

We presented a case of an EBV-related conjunctival mass in a paediatric patient with a recent primary infection and symptoms of infectious mononucleosis. The blood picture was not fully characteristic and the instance emphasised the importance of recognising the early signs of IM and treating accordingly. Ultimately, surgical excision was performed and the child had a successful recovery shortly after.

## Consent

Written informed consent was obtained from the patient’s parents for publication of this Case report and any accompanying images. A copy of the written consent is available for review by the Editor of this journal.
